# Determining a relative total lumbar range of motion to alleviate adjacent segment degeneration after transforaminal lumbar interbody fusion: a finite element analysis

**DOI:** 10.1186/s12891-024-07322-3

**Published:** 2024-03-05

**Authors:** Ke Li, Shuai Cao, Jing Chen, Jie Qin, Bo Yuan, Jie Li

**Affiliations:** 1 Department of Orthopedics, The Second Affiliated Hospital of Xi’an Jiaotong University, 157th West Fifth Road, Xi’an, Shaanxi Province 710004 China; 2 Department of Orthopedics, Civil Aviation General Hospital, No. 1, Gaojing Stress, Chaoyang District, Beijing, 100123 China

**Keywords:** Range of motion, Adjacent segment degeneration, Finite element analysis

## Abstract

**Background:**

A reduction in total lumbar range of motion (ROM) after lumbar fusion may offset the increase in intradiscal pressure (IDP) and facet joint force (FJF) caused by the abnormally increased ROM at adjacent segments. This study aimed to determine a relative total lumbar ROM rather than an ideal adjacent segment ROM to guide postoperative waist activities and further delay adjacent segment degeneration (ASD).

**Methods:**

An intact L1-S1 finite element model was constructed and validated. Based on this, a surgical model was created to allow the simulation of L4/5 transforaminal lumbar interbody fusion (TLIF). Under the maximum total L1-S1 ROM, the ROM, IDP, and FJF of each adjacent segment between the intact and TLIF models were compared to explore the biomechanical influence of lumbar fusion on adjacent segments. Subsequently, the functional relationship between total L1-S1 ROM and IDP or total L1-S1 ROM and FJF was fitted in the TLIF model to calculate the relative total L1-S1 ROMs without an increase in IDP and FJF.

**Results:**

Compared with those of the intact model, the ROM, IDP, and FJF of the adjacent segments in the TLIF model increased by 12.6-28.9%, 0.1-6.8%, and 0-134.2%, respectively. As the total L1-S1 ROM increased, the IDP and FJF of each adjacent segment increased by varying degrees. The relative total L1-S1 ROMs in the TLIF model were 11.03°, 12.50°, 12.14°, and 9.82° in flexion, extension, lateral bending, and axial rotation, respectively.

**Conclusions:**

The relative total L1-S1 ROMs after TLIF were determined, which decreased by 19.6-29.3% compared to the preoperative ones. Guiding the patients to perform postoperative waist activities within these specific ROMs, an increase in the IDP and FJF of adjacent segments may be effectively offset, thereby alleviating ASD.

## Background

Low back pain (LBP) resulting from lumbar pathologies, such as lumbar disc herniation and lumbar spondylolisthesis, is a globally prevalent issue and is recognized as a leading cause of productivity loss [1]. The majority of individuals experience at least one episode of acute LBP during their lifetime, with a considerable proportion transitioning into a chronic condition. Studies indicated that the prevalence of chronic LBP can reach as high as 57% [2]. It is intricately associated with lumbar segmental instability, encompassing intervertebral disc, ligament, facet joint, and muscle dysfunctions [3]. When the patients experienced poor therapeutic effects as a result of conservative treatment including anti-inflammatory medications, physical therapy, and local corticosteroid injections, the operation became a suitable scheme [4]. At present, lumbar interbody fusion combined with pedicle screw fixation is most commonly used to achieve reduction, decompression, fixation, and fusion [5]. Although the concept and efficacy of the operation have been widely recognized, consequent adjacent segment degeneration (ASD) remains a troublesome complication after surgery. Some studies reported that the incidence of radiographic and symptomatic ASD after lumbar fusion was 26.6% and 8.5%, respectively [6]. Additionally, Lee et al. reported that 28 of the 1069 patients (2.62%) who were included in the study needed secondary operations due to ASD [7]. Therefore, mitigating postoperative ASD is a serious challenge.

Currently, it is generally believed that ASD is a complex pathophysiological process caused by multiple factors. There is solid evidence that alterations in range of motion (ROM), intradiscal pressure (IDP), and facet joint force (FJF) are closely related to the occurrence and development of this complication [8]. The finite element study by Du et al. also revealed that the ROM, IDP, and FJF of adjacent segments increased after lumbar interbody fusion because the pattern of motion and load distribution had been modified [9]. The reduced ROM of the fixed segment is compensated by other segments to restore the overall motor function of the spine, which can lead to an abnormal increase in IDP and FJF [10]. Thus, the published literature has mainly focused on reducing ROM, IDP, and FJF to alleviate ASD by improving surgical techniques, such as semirigid fixation and dynamic fixation [11–13]. However, few studies have addressed this issue from the perspective of postoperative waist activities.

This study is based on the following hypothesis: With a decrease in total L1-S1 ROM, the ROM of each adjacent segment will decrease correspondingly, which can offset the increase in IDP and FJF after lumbar fusion [14]. Based on this hypothesis, a relative total L1-S1 ROM without an increase in IDP and FJF was proposed to guide the patients to conduct postoperative waist activities, which may help delay the progression of ASD. Therefore, the purpose of this study is to find an ideal total L1-S1 ROM rather than an ideal adjacent segment ROM.

To this end, an intact finite element model of L1-S1 was constructed, and a surgical model of L4/5 transforaminal lumbar interbody fusion (TLIF) was simulated. To explore the biomechanical effects of lumbar fusion on adjacent segments, the ROM, IDP, and FJF of each adjacent segment between the intact and TLIF models under the maximum total L1-S1 ROM were compared. Then, the functional relationship between total L1-S1 ROM and IDP or total L1-S1 ROM and FJF was fitted in the TLIF model. Finally, the relative total L1-S1 ROMs without an increase in IDP and FJF were calculated using the above fitting functions.

## Methods

### Construction of intact finite element model

A healthy male (30 years of age; height, 176 cm; weight, 60 kg) without lumbar disease was selected to undergo lumbar thin-layer (0.625 mm) CT scanning. This study was approved by the Ethics Committee of the Second Affiliated Hospital of Xi’an Jiaotong University, and informed consent was obtained from the subject. Then, the images were imported into Mimics 20.0 (Materialise, Leuven, Belgium) to construct a L1-S1 bone surface model that was optimized by noise reduction, smoothing, and cavity filling. Next, solid models of the vertebral cortical shell, cancellous bone, and intervertebral disc were constructed using 3-Matic 11.0 (Materialise, Leuven, Belgium). After that, the model was imported into HyperMesh 14.0 (Altair Engineering, Inc., Troy, Michigan, USA) for a series of preprocessing operations for finite element analysis, including model assembly, ligament construction, material property definitions, and meshing. Finally, Abaqus 6.13 (Dassault System, Paris, France) was used as the solver for the boundary condition set, finite element analysis, and postprocessing.

**Fig. 1:**
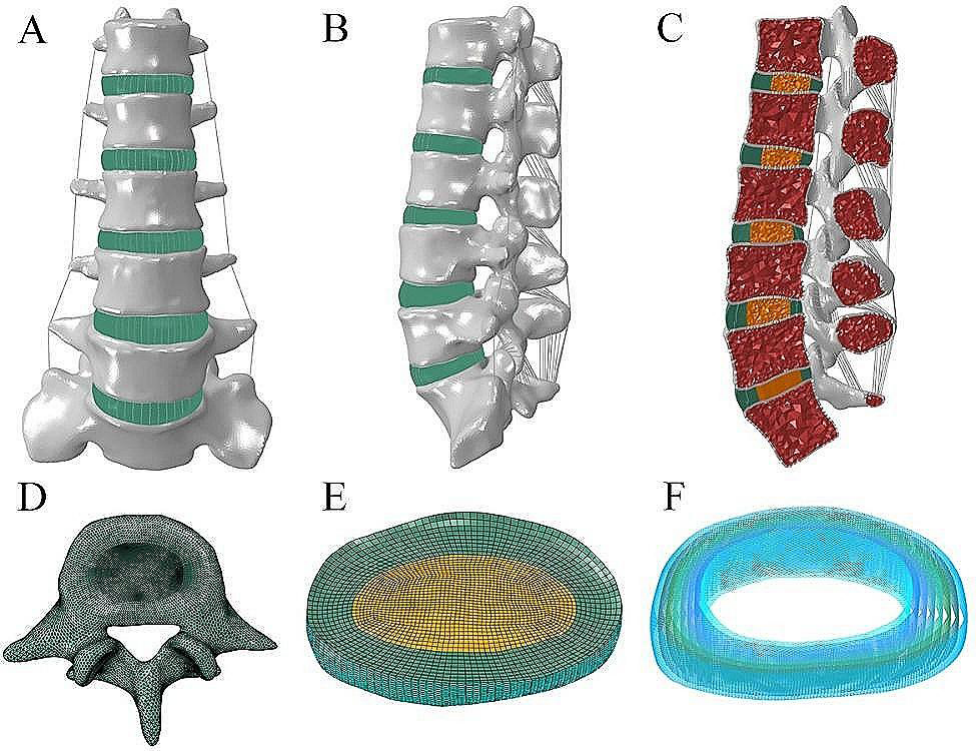
The intact finite element model. (**A**) Front view. (**B**) Lateral view. (**C**) Longitudinal section. (**D**) Top view. (**E**) Intervertebral disc. (**F**) Annulus fiber

The intact model contained 6 vertebrae, 5 intervertebral discs, and 7 ligaments (Fig. 1A-D). The specific modeling methods referenced previous studies [15–17]. 1-mm-thick cortical layers and bony endplates surrounded the cancellous bone. The cartilage endplates with a thickness of 0.5 mm covered the surface of the bony endplates [18]. The nucleus pulposus accounted for approximately 30-40% of the intervertebral disc volume (Fig. 1E). Eight layers of fibers were embedded in the ground substance in concentric rings around the nucleus pulposus (Fig. 1F). The fibers were oriented at an angle of ± 30°-45° from the horizontal surface defined by the bottoms of intervertebral discs. From the outermost (550 MPa) to the innermost (360 MPa) layer, the elastic strength decreased proportionally to ensure the variations in fiber stiffness. According to the anatomical position of ligaments, seven kinds of ligaments were constructed. All ligaments were modeled as tension truss elements. Facet cartilage joints were modeled as frictionless soft contacts. To ensure that the strain energy changes did not exceed 5%, a convergence analysis was performed. The element types and material properties are shown in Table 1.

**Table 1:** Materials properties and element types in the finite element models

Materials	Element type	Young’s modulus (MPa)	Poisson’s ratio (µ)
Cortical bone	C3D4	12,000	0.3
Cancellous bone	C3D4	100	0.2
Bony endplate	C3D8I	1,200	0.29
Cartilage endplate	C3D8I	24	0.4
Nucleus pulposus	C3D8H	C10 = 0.12, C01 = 0.09, D = 0	
Annulus ground	C3D8H	C10 = 0.1333, C01 = 0.0333, D = 0.6	
Annulus fiber	T3D2	360–550	
Anterior longitudinal	T3D2	7.8 (< 12), 20 (> 12%)	
Posterior longitudinal	T3D2	10 (< 11%), 20 (> 11%)	
Ligamentum flavum	T3D2	15 (< 6.2%), 19.5 (> 6.2%)	
Supraspinous	T3D2	8.0 (< 20%), 15 (> 20%)	
Interspinous	T3D2	10 (< 14%), 11.6 (> 14%)	
Intertransverse	T3D2	10 (< 18%), 58.7 (> 18%)	
Capsular	T3D2	7.5 (< 25%), 32.9 (> 25%)	
Screws and rods	C3D4	110,000	0.3
Cage	C3D8	3,600	0.25
Bone grafts	C3D8	100	0.2

### Finite element modeling of L4/5 TLIF procedures

**Fig. 2:**
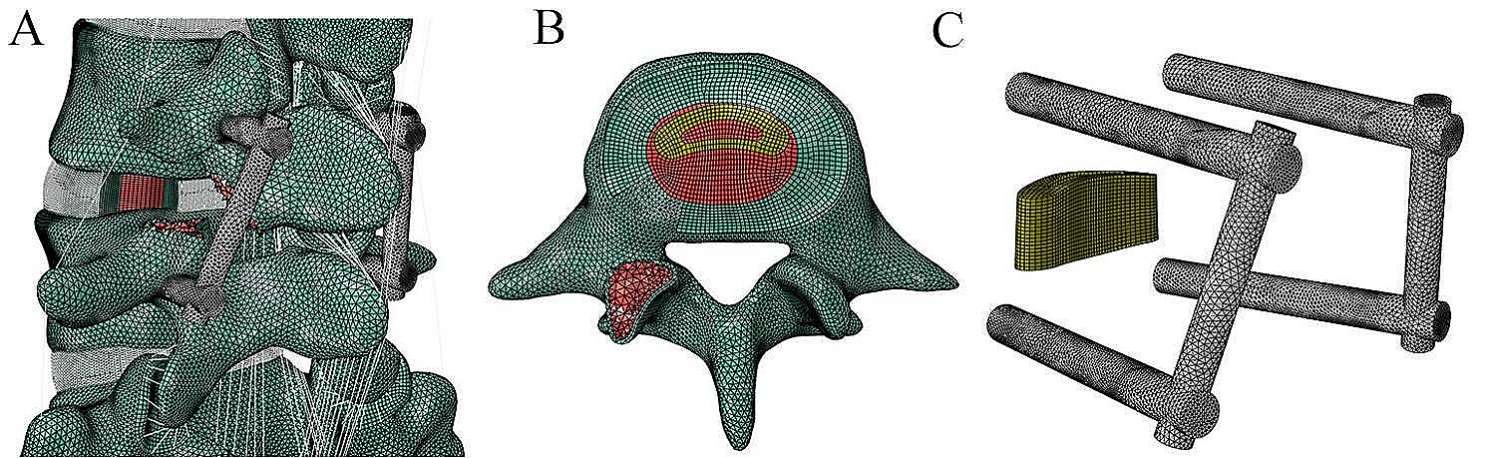
The surgical model for transforaminal lumbar interbody fusion. (**A**) Posterior oblique view. (**B**) Cage and bone grafts. (**C**) Screw-rod system and cage

The TLIF model was built as reported previously in the literature [19]. To simulate the processes of decompression and fusion, a left L4/5 facetectomy was performed; then, the entire nucleus pulposus, the left posterior part of the annulus fibrosus, and capsular and flavum ligaments were removed (Fig. 2A). The central region and the left posterior portion of the cartilage endplate at the L4/5 segment were removed to simulate the endplate preparation. A PEEK cage was placed on the anterior part of the L4/5 intervertebral space (Fig. 2B). Cancellous bone was implanted into the inner and outer spaces of the cage to fill the intervertebral space. The pedicle screw-based fixation system consisted of four screws (diameter, 6.5 mm; length, 45 mm) and two connecting rods (diameter, 5.5 mm; length, 58 mm) (Fig. 2C). The cage-bone graft, cage-endplate, bone graft-endplate, bone-screw, and screw-rod interfaces were assigned a “tie” constraint.

### Boundary and loading conditions

The inferior surface of the S1 vertebra for the model was constrained. A 400 N follower load was applied to the L1 superior surface to simulate the upper torso of a normal adult and muscle force. The load path was consistent with the physiological curvature of the lumbar spine. For the intact model, an additional moment of 8 Nm was applied to the L1 vertebra to produce flexion, extension, lateral bending, and axial rotation. To validate the intact model, the segmental ROM under the combined loading modes consisting of the moment and follower load was compared with previously reported values [20–23].

Under the same follower load, the TLIF model was subjected to displacement loads to ensure that its maximum total L1-S1 ROM was similar to those of the intact model. To investigate the biomechanical impact of lumbar fusion on adjacent segments, the ROM, IDP, and FJF of each adjacent segment between the intact and TLIF models under the maximum total L1-S1 ROM were compared. To reflect the functional relationship between total L1-S1 ROM and IDP or total L1-S1 ROM and FJF, the IDP or FJF values at the adjacent segments were recorded under different L1-S1 ROMs. Based on these limited numerical points, continuous functions were obtained. The total L1-S1 ROM corresponding to specific IDP or FJF value can be calculated through these functions.

## Results

### Model validation

**Fig. 3:**
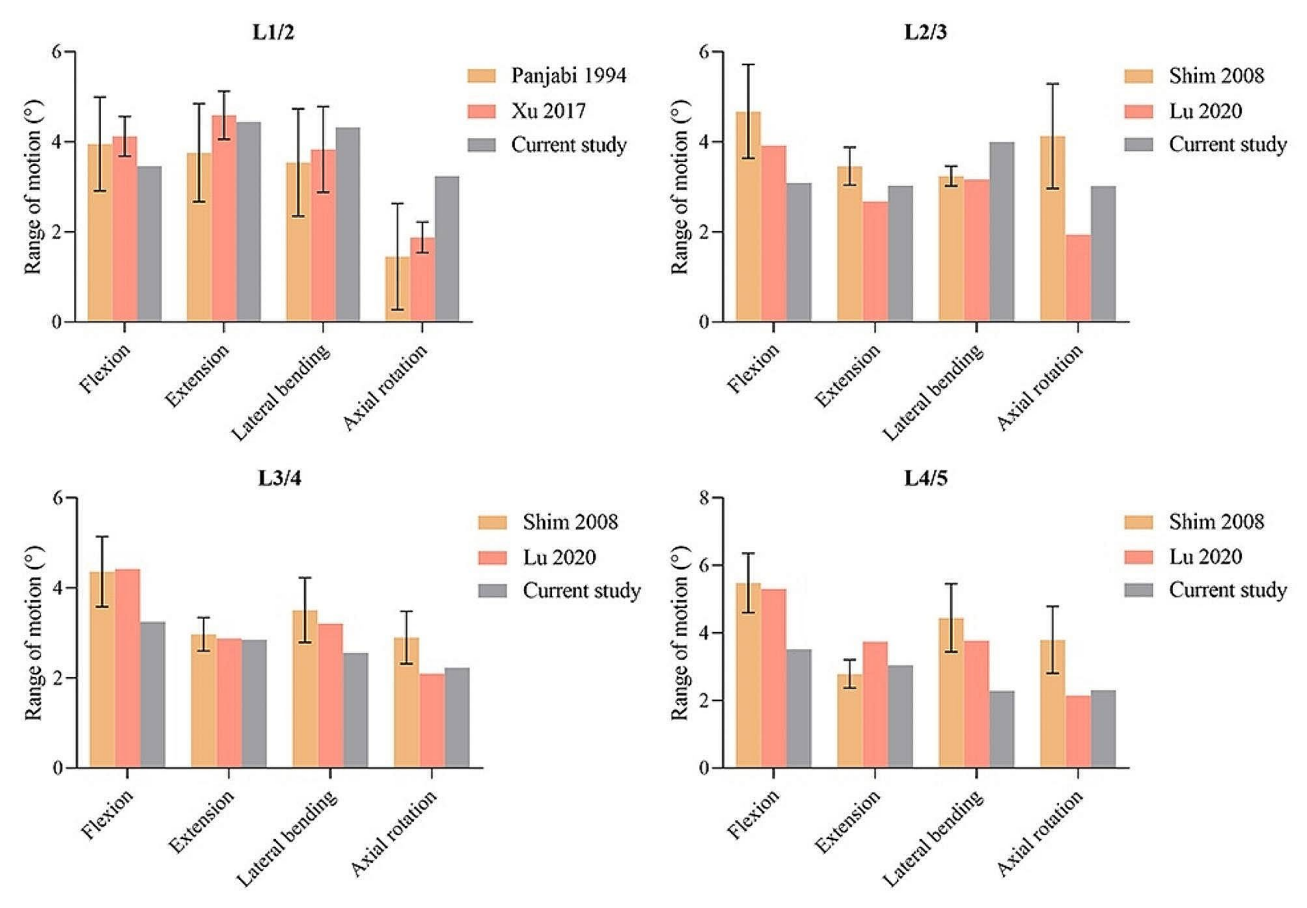
Comparison of the range of motion at each segment between the current and previous studies

To validate the reliability of the intact model, the ROM of each segment under combined loading modes of 8 Nm moment and 400 N follower load was calculated. The simulation results were compared with in vivo experimental data and results predicted by the finite element models in the literature [20–23]. Together, these results demonstrated that the simulation showed good quantitative agreement with the in vitro data (Fig. 3). Consequently, the current model was reliable and suitable for further research.

### ROM

**Fig. 4:**
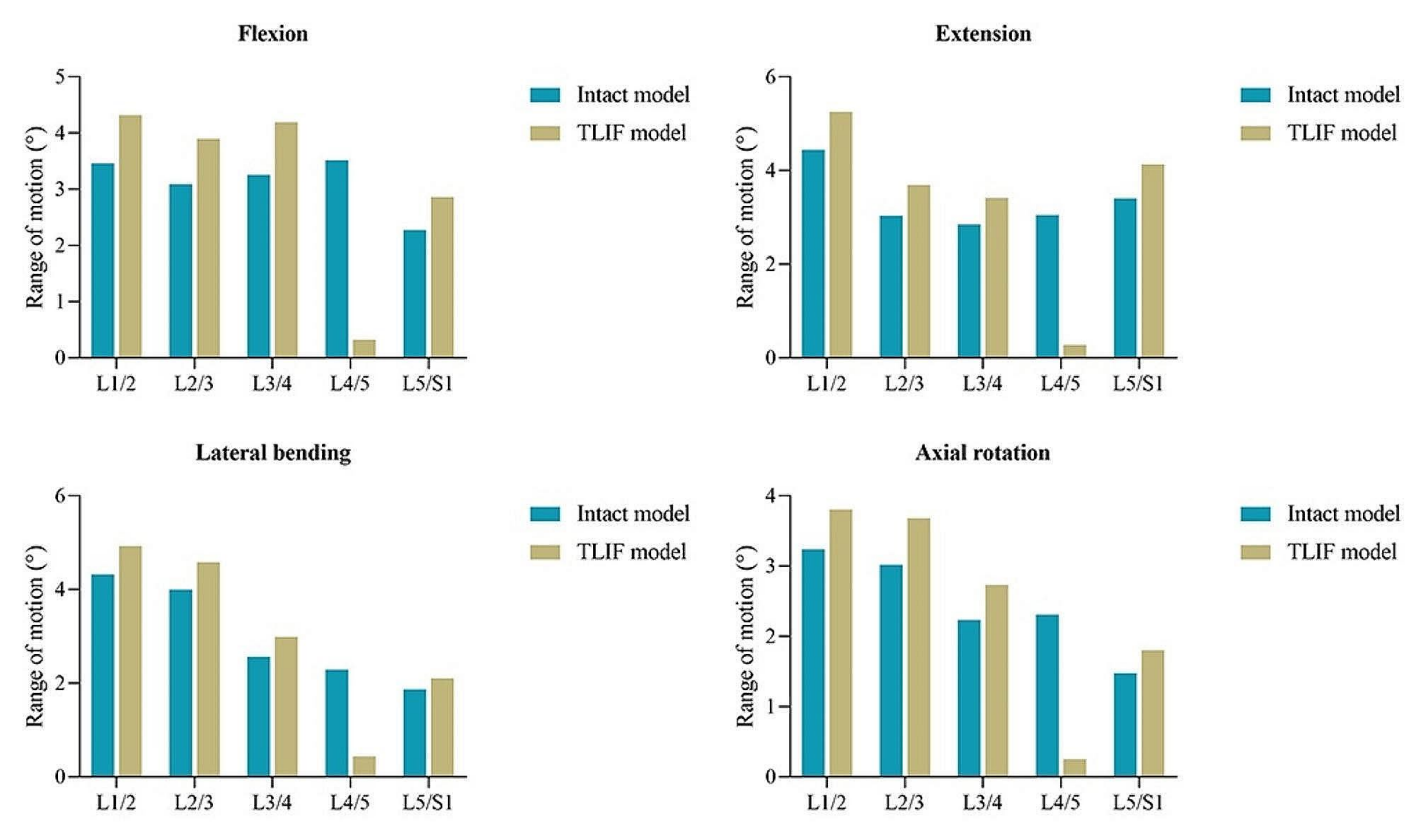
Comparison of the ROM at each segment between the intact and TLIF models under the maximum total L1-S1 ROM. ROM: range of motion; TLIF: transforaminal lumbar interbody fusion

Under the maximum total L1-S1 ROM, the ROMs of the L4/5 segment in the TLIF model were 0.32°, 0.28°, 0.44°, and 0.25° in flexion, extension, lateral bending, and axial rotation, respectively. Compared with that of the intact model, the L4/5 ROM of the TLIF model decreased by 80.8-90.9%. During flexion, extension, lateral bending, and axial rotation, the ROMs in the TLIF model were 4.32°, 5.25°, 4.93°, and 3.80° at the L1/2 segment; 3.90°, 3.69°, 4.58°, and 3.68° at the L2/3 segment; 4.20°, 3.41°, 2.99°, and 2.73° at the L3/4 segment; and 2.86°, 4.13°, 2.10°, and 1.80° at the L5/S1 segment, respectively. Compared with the intact model, the ROM of the TLIF model increased by 14.0-24.8% at the L1/2 segment, 14.7-26.1% at the L2/3 segment, 16.7-28.9% at the L3/4 segment, and 12.6-25.8% at the L5/S1 segment. Overall, the ROMs of the adjacent segments in the TLIF model were higher than those in the intact model, and the increase was the largest in flexion and the smallest in lateral bending (Fig. 4).

### IDP

**Fig. 5:**
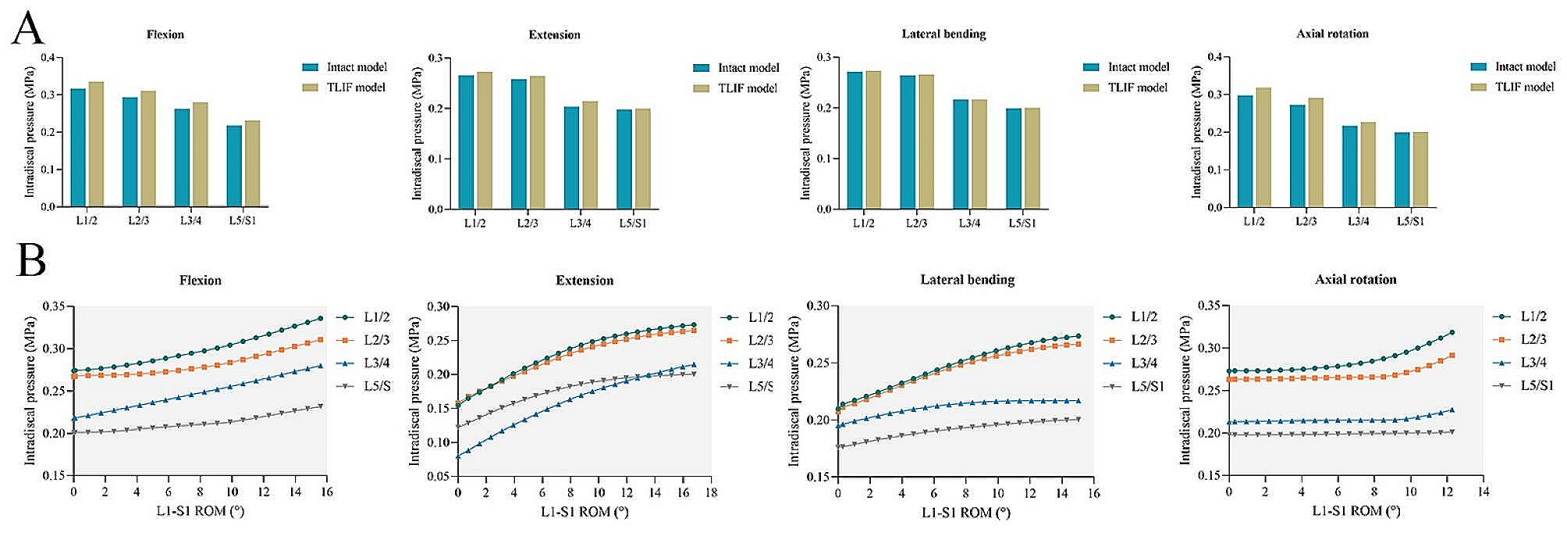
The changes in intradiscal pressure at the adjacent segments. (**A**) The maximum intradiscal pressure in the intact and TLIF models. (**B**) Changes in intradiscal pressure with the total L1-S1 ROMs in the TLIF model. ROM: range of motion; TLIF: transforaminal lumbar interbody fusion

During flexion, extension, lateral bending, and axial rotation, the maximum IDP values in the TLIF model were 0.34 MPa, 0.27 MPa, 0.27 MPa, and 0.32 MPa at the L1/2 segment, respectively; 0.31 MPa, 0.26 MPa, 0.27 MPa, and 0.29 MPa at the L2/3 segment, respectively; 0.28 MPa, 0.21 MPa, 0.22 MPa, and 0.23 MPa at the L3/4 segment, respectively; and 0.23 MPa, 0.20 MPa, 0.20 MPa, and 0.20 MPa at the L5/S1 segment, respectively. Compared with the intact model, the maximum IDP of the TLIF model increased by 0.7-6.8% at the L1/2 segment, 0.8-6.8% at the L2/3 segment, 0.1-6.6% at the L3/4 segment, and 0.5-6.4% at the L5/S1 segment (Fig. 5A). With the increase in total L1-S1 ROMs, the IDP of each adjacent segment increased in all motion directions (Fig. 5B). The relationship between IDP and total L1-S1 ROM was synthesized into continuous function.

#### FJF

**Fig. 6:**
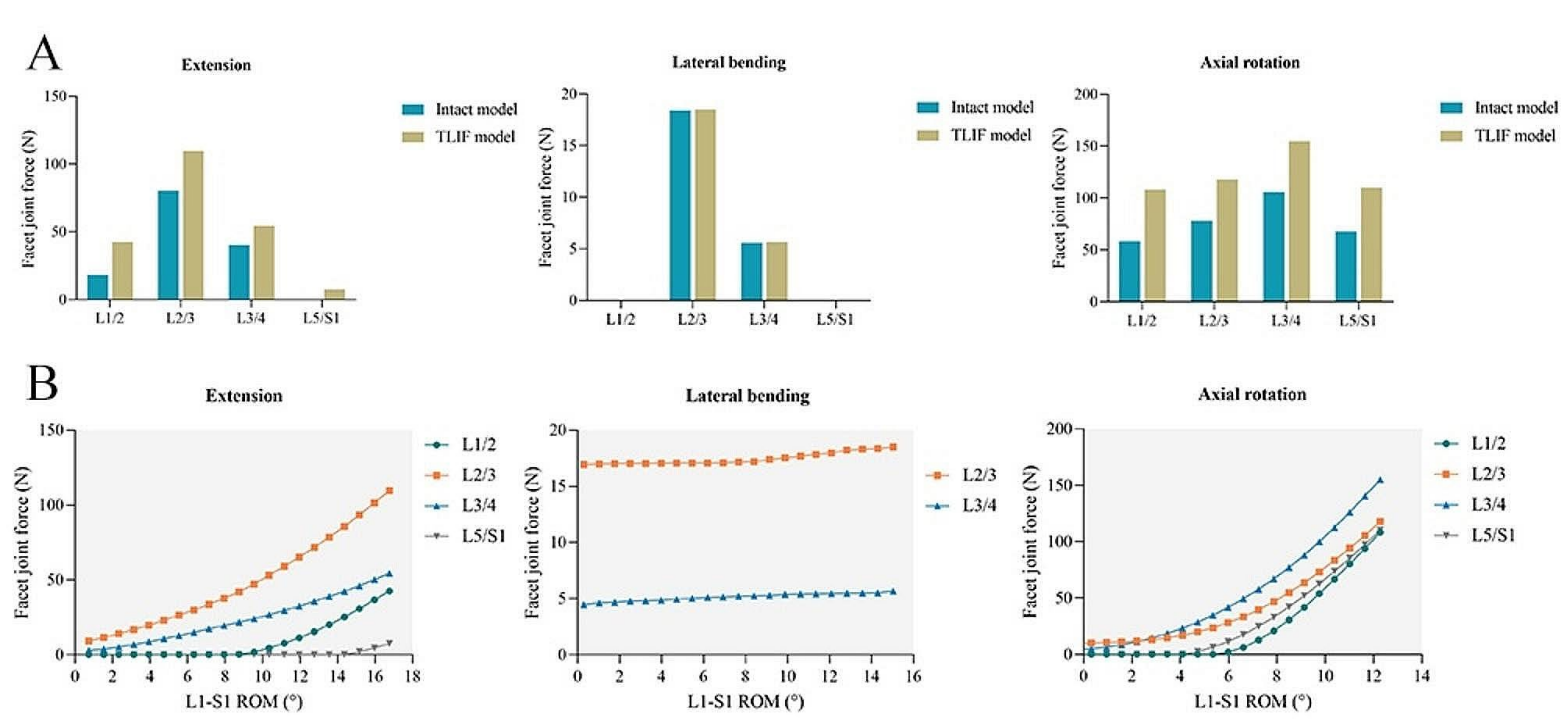
The changes in facet joint force at the adjacent segments. (**A**) The maximum facet joint force in the intact and TLIF models. (**B**) Changes in facet joint force with the total L1-S1 ROMs in the TLIF model. ROM: range of motion; TLIF: transforaminal lumbar interbody fusion

During flexion, the FJF values of each segment were not obtained because the facet joints were not in contact. For lateral bending, the FJF values of the L1/2 and L5/S1 segments were not measured. In addition, the maximum FJF values of the intact model and TLIF model at the L2/3 segment (18.43 N vs. 18.52 N) and L3/4 segment (5.57 N vs. 5.68 N) were similar during lateral bending. During extension and axial rotation, the maximum FJF values in the TLIF model were 42.59 and 108.25 N at the L1/2 segment, respectively; 109.66 and 117.84 N at the L2/3 segment, respectively; 54.42 and 155.00 N at the L3/4 segment, respectively; and 7.46 and 110.21 N at the L5/S1 segment, respectively. Compared with the intact model, the maximum FJF of the TLIF model during extension and axial rotation increased by 85.2-134.2% at the L1/2 segment, 36.4-50.7% at the L2/3 segment, 35.5-46.5% at the L3/4 segment, and 62.8% at the L5/S1 segment (Fig. 6A). With the increase in total L1-S1 ROMs, the FJF of each adjacent segment increased (Fig. 6B). The relationship between FJF and total L1-S1 ROM was synthesized into continuous function.

### Relative total L1-S1 ROMs after L4/5 TLIF

**Fig. 7:**
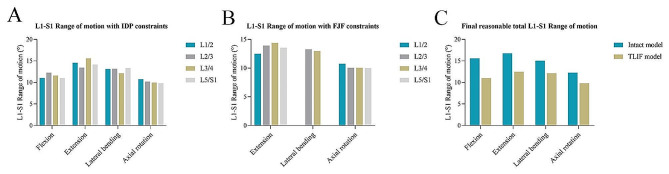
Determination of a relative total lumbar ROM. (**A**) The total L1-S1 ROM with IDP constraints. (**B**) The total L1-S1 ROM with FJF constraints. (**C**) The relative total L1-S1 ROM in the TLIF model. ROM: range of motion; IDP: intradiscal pressure; FJF: facet joint force; TLIF: transforaminal lumbar interbody fusion

The total L1-S1 ROM with IDP or FJF constraint in the TLIF model was calculated using the fitting functions when the IDP or FJF value in the TLIF model was the same as the maximum IDP or FJF value in the intact model. The total L1-S1 ROMs with IDP and FJF constraints in each segment and direction were shown in Fig. 7A-B. The relative total L1-S1 ROM was determined as the minimum total L1-S1 ROMs with IDP and FJF constraints in all adjacent segments. As a result, the relative total L1-S1 ROMs were 11.03°, 12.50°, 12.14°, and 9.82° in flexion, extension, lateral bending, and axial rotation, respectively. Compared with the intact model, the relative total L1-S1 ROMs of the TLIF model decreased by 29.3%, 25.5%, 19.6%, and 19.9% in flexion, extension, lateral bending, and axial rotation, respectively (Fig. 7C).

## Discussion

ASD after lumbar fusion is a common and important long-term complication. Due to the long course of the disease and the complexity of its etiology, the incidence of ASD and its reoperation rate varied widely in previous reports [24, 25]. Although its true incidence is still unclear, measures to delay this complication are essential to improve the patients’ prognoses. This study attempted to explore the biomechanical changes of the adjacent segments after TLIF and to determine a relative total ROM of the lumbar spine to alleviate segmental degeneration and guide patients during moderate waist activities. Our study found that compared to the intact model, the L4/5 TLIF model increased the ROM, IDP, and FJF in all the adjacent segments. As the total L1-S1 ROM increased, the IDP and FJF increased by varying degrees. Finally, relative total L1-S1 ROMs that did not increase the IDP and FJF at adjacent segments were determined, which were 19.6-29.3% lower than preoperative ROMs.

Although some scholars believe that ASD is a natural course unrelated to fusion, an overwhelming body of literature supports the view that surgery changes the biomechanics of adjacent segments and further promotes segmental degeneration [26, 27]. Sacrificing the ROM of the responsible segment to achieve spinal stability is a basic concept of lumbar fusion. To restore the total ROM of the lumbar spine to ensure that daily activities are not restricted as much as possible, the reduced ROM is often compensated by other segments [28, 29]. This compensatory mechanism may be accompanied by a change in the lever arm or center of motion, which further leads to an increase in IDP and FJF [30]. Moreover, the implantation of instrumentation, as in screw-rod fixation, that does not match the elastic modulus of human bone tissue alters the load transmission of the spine, which may increase the stress rate and load of adjacent segments, resulting in structural damage and mechanical failure of intervertebral discs and facet joints [31, 32]. At the cellular level, an increase in IDP and FJF induces a series of consequences on the biochemical environment of the intervertebral disc and facet joints [33, 34]. The abnormal release of cytokines such as interleukin-1b and tumor necrosis factor-α triggers the inflammatory cascade and matrix remodeling [35, 36]. Furthermore, hypoxia, autophagy, apoptosis, and other mechanisms are responsible for intervertebral disc degeneration and facet joint osteoarthritis [37].

Interestingly, our findings indicated that the biomechanical changes in adjacent segments near the fixed segment were not significantly greater than those in other segments. In other words, the compensatory mechanism of the adjacent segment to ROM and changes in IDP and FJF may occur evenly in each segment. This result is consistent with the views of Kolstad et al. [38–40]. We thought that the occurrence and development of ASD should depend on the motion characteristics of each segment and its adaptability to load changes, rather than the distance from the fixed segment [41]. More importantly, different segments may have been in different stages of degeneration before the surgical intervention. Thus, which segment is most prone to degeneration after lumbar fusion is an unpredictable problem. Additionally, it is generally believed that intervertebral disc degeneration and facet joint degeneration affect each other and together lead to segmental degeneration. However, which one has a greater impact on ASD remains unknown. Our study found that the IDP of adjacent segments increased slightly in all motion directions after lumbar fusion; however, FJF significantly increased during extension and axial rotation. This might indicate that facet joint degeneration plays a greater role than disc degeneration in the process of ASD. The study by Lee et al. also showed that facet degeneration was a significant risk factor for ASD, rather than disc degeneration [7].

Numerous studies have been conducted to investigate the factors influencing ASD. Wangsawatwong et al. found that lateral lumbar interbody fusion results in significantly reduced mobility of adjacent segments compared to TLIF and posterior lumbar interbody fusion [42]. They also revealed that cortical screw–rod fixation or pedicle screw–rod fixation can significantly affect the adjacent segment motion [43]. According to Zhao et al., adjacent segment ROM increased with decreased lumbar lordosis of fused levels [44]. Poor sagittal balance, however, was only a limited risk factor for ASD revisions in the study by Toivonen et al. [45]. To reduce the risk of ASD, researchers have developed many dynamic and semirigid fixation devices to preserve spinal movement and distribute the load reasonably [46, 47]. However, the effectiveness of these devices has been questioned. As the main results of this study, we determined a relative total lumbar ROM after L4/5 TLIF. These findings offer some guidance to patients in engaging in appropriate waist activities and preventing excessive lumbar ROM after surgery, thereby potentially slowing down the advancement of ASD.

This study has some limitations. First, there is a big gap between the model and the real conditions in vivo, because the muscle, nerve, blood vessels, and other structures can not be simulated effectively. Second, IDP and FJF were used to evaluate the risk of ASD in our study. However, ASD is a complex process involving multiple risk factors, such as age, body mass index, sagittal vertical axis, pelvic incidence, number of fusion segments, and facet joint violation [48]. Thus, further studies are required to validate our results. Finally, the intact model was built based on lumbar CT images obtained from a young, healthy volunteer. Therefore, the extrapolation of the conclusion should be scrupulous.

## Conclusion

L4/5 TLIF increased the ROM, IDP, and FJF of the adjacent segments compared with before surgery. As the total L1-S1 ROM increased, the IDP and FJF of each adjacent segment increased by varying degrees. The relative total L1-S1 ROMs after TLIF were determined, which decreased by 19.6-29.3% compared to the preoperative ROMs. Guiding the patients to perform postoperative waist activities within these specific ROMs, an increase in the IDP and FJF of adjacent segments may be effectively offset, thereby alleviating ASD.

## Data Availability

All data generated or analyzed are included in this article.

## Abbreviations

LBP: low back pain
ROM: range of motion
FJF: facet joint force
IDP: intradiscal pressure
ASD: adjacent segment degeneration
TLIF: transforaminal lumbar interbody fusion
